# Effects of Acid Rock Drainage on Microbial Communities in Alpine Streams of the Pyrenees

**DOI:** 10.1007/s00248-025-02667-1

**Published:** 2025-12-05

**Authors:** José Luis Guijosa-Ortega, Anna M. Romaní, Oriol Grau, Sergi Pla-Rabés, Olga Margalef, José Gabriel Salminci, Mario Zarroca, Ada Pastor

**Affiliations:** 1 GRECO, Institute of Aquatic Ecology, University of Girona, Girona, Spain; 2 Parc Natural de l’Alt Pirineu, Llavorsí, 25595 Lleida Spain; 3 BABVE, Autonomous University of Barcelona, Cerdanyola del Vallès, Barcelona, Spain; 4 CSIC-CREAF, Cerdanyola del Vallès, Barcelona, Spain; 5 RISKNAT Research Group, Department of Earth and Ocean Dynamics, University of Barcelona, Barcelona, Spain; 6 External Geodynamics and Hydrogeology Group, Autonomous University of Barcelona, Cerdanyola del Vallès, Barcelona, Spain

**Keywords:** PH, Freshwater, Bacteria, Eukaryotes, Diatoms, Indicative taxa

## Abstract

Weathering of sulphur-bearing rocks leads to acid rock drainage (ARD), which decreases water pH, mobilizes heavy metals, and forms coloured coatings of metal precipitates on riverbeds. This study assessed the effects of ARD on microbial biofilm biodiversity and community structure in alpine streams across two Pyrenean regions (Núria and Chistau). Biofilms were sampled from acidic (pH < 5.5) and non-acidic (pH > 6.5) streams, and at their confluence, where metal precipitates occur (white-coated streams). We characterised bacterial and eukaryote communities by molecular tools and specifically analysed the diatom communities by morphology approach. Their respective community composition varied with stream category for both bacteria and eukaryotes, but only bacteria exhibited a loss in diversity in acidic and white-coated streams. Diatom communities and diversity differences were driven mainly by region. In acidic and white-coated streams, bacteria which can use metals and sulphurs in their metabolic processes increased, together with fungi and some photosynthetic groups (*Chlorophyta*, *Streptophyta*) among eukaryotes. Amplicon Sequence Variants (ASVs) assigned to acidophilic and psychrotolerant bacteria were highly associated with acidic streams, and *Cyanophyceae* ASVs were highly associated with white-coated ones. As for eukaryotes, ASVs of *Chrysophyceae* were associated with both acidic and white-coated streams. Nonetheless, the regional factor remained consistently significant across microbial communities. This study indicates that ARD-affected streams can support microbial communities adapted to their extreme conditions, with the communities in white-coated rivers differing markedly from those in acidic rivers.

**Supplementary Information:**

The online version contains supplementary material available at 10.1007/s00248-025-02667-1.

## Introduction

Rock weathering can cause acid rock drainage (ARD), which occurs when sulphur-bearing rocks are exposed to water flow and/or to the atmosphere. Weathering of rocks containing pyrite (FeS_2_) and other easily oxidizable sulphides, forms sulphuric acid, resulting in low pH and sulphur export [1]. Acidic waters accelerate the weathering of other rocks and minerals, releasing both essential and toxic metals, which can be harmful even at low concentrations, such as arsenic [2]. These metals and sulphates can precipitate forming coloured coatings on the streambed due to the high pH gradient in downstream confluences, where acidic and near-neutral waters mix [3].

The natural ARD phenomenon has been documented in pristine alpine regions worldwide, including the Peruvian Andes [4], Rocky Mountains [5], Alps [6] and Pyrenees [7]. In headwaters of Central Colorado’s Rocky Mountains, dissolved metals and SO_4_^2−^ concentrations increased over 30 years, coinciding with rising temperatures and occurring independently of mining impacts [5]. In the Alps, rock glaciers have been associated with ARD, as melting of ice and snowpacks expose mineral-rich debris containing high metal concentrations [6]. In the Pyrenees, prolonged drought and warming have been linked with the formation of white-coloured streambeds made of aluminium hydroxides. Reduced precipitation may reduce the dilution of solute-rich flow. Meanwhile, higher air temperatures could increase the exposure of fresh rock surface to oxidation by reducing snow and ice cover, as well as accelerating sulphide bio-chemical oxidation through its positive feedback with temperature [7]. While these studies predominantly focus on geological and hydrochemical aspects, they offer limited insight into ARD´s biological implications in freshwater ecosystems.

In alpine streams, microbial biofilms are closely connected to their surrounding environment, taking up, retaining, and transforming organic compounds, nutrients and metals. These biofilms consist of communities of bacteria, algae, protozoa and fungi embedded in a polysaccharide matrix, which live attached to multiple submerged substrates on the streambed [8, 9]. Due to their crucial role in biogeochemical cycling, ubiquity, rapid response time, and trophic relevance, microbial biofilms can serve to detect the early effects of changes in environmental conditions on the ecosystem [10]. Indeed, bacterial and eukaryotic communities have been shown to be responsive to different pH conditions and metal concentrations [11]. Among all eukaryotic groups, diatom communities have commonly been used as freshwater quality bioindicators due to their high sensitivity to environmental changes [12]. However, studies generally show that metals and low pH reduce bacterial, eukaryotic and specifically diatom diversity, although specialist taxa may thrive in such extreme environments [13, 14]. However, most research focuses on metals and pH effects in relation to mining and industrial activities [15], limiting our understanding of their impact in natural ARD settings.

Despite similar acidification processes, microbial responses to natural ARD may differ from those caused by mining activity. Naturally acidic waters in these alpine ecosystems originate from the oxidation of sub-surface and underground mineral deposits. Thereby, their exposure to atmospheric oxidative conditions and acidification rates are relatively limited [16]. In addition, these natural alpine acidic environments, despite being scarce, have persisted long enough for host communities to develop evolutionary adaptations to natural acidification [16, 17]. Meanwhile, anthropogenic acidification normally comes from massive expositions of mineral debris to the atmosphere, being able to change the metal speciation and acidifying waters faster and with more intensity, causing perturbations and changes in microbial communities [16, 18]. However, few studies have examined the ecological effects of natural ARD on microbial biofilms in these pristine, vulnerable ecosystems, revealing a significant knowledge gap that requires further research.

The main objective of this study was to evaluate the effects of ARD on the diversity and composition of the microbial biofilm communities in alpine streams of the Pyrenees. Specifically, we aimed to: (1) determine how ARD affects the alpha diversity of stream microbial biofilm communities, (2) assess how it impacts their community composition, and (3) identify potential indicative taxa of ARD effects. We hypothesized that ARD extreme conditions, like low pH and high metal concentrations, would alter biofilm alpha diversity and community composition compared to non-affected ecosystems. To test this, we characterized bacterial, eukaryotic, and specifically diatom communities in ARD-affected streams (acidic, white-coated) and non-affected in two Pyrenean valleys. This study aims to shed light on the ecological and biological effects of natural ARD on alpine stream ecosystems.

## Methods

### Study Area and Sampling Strategy

**Fig. 1:**
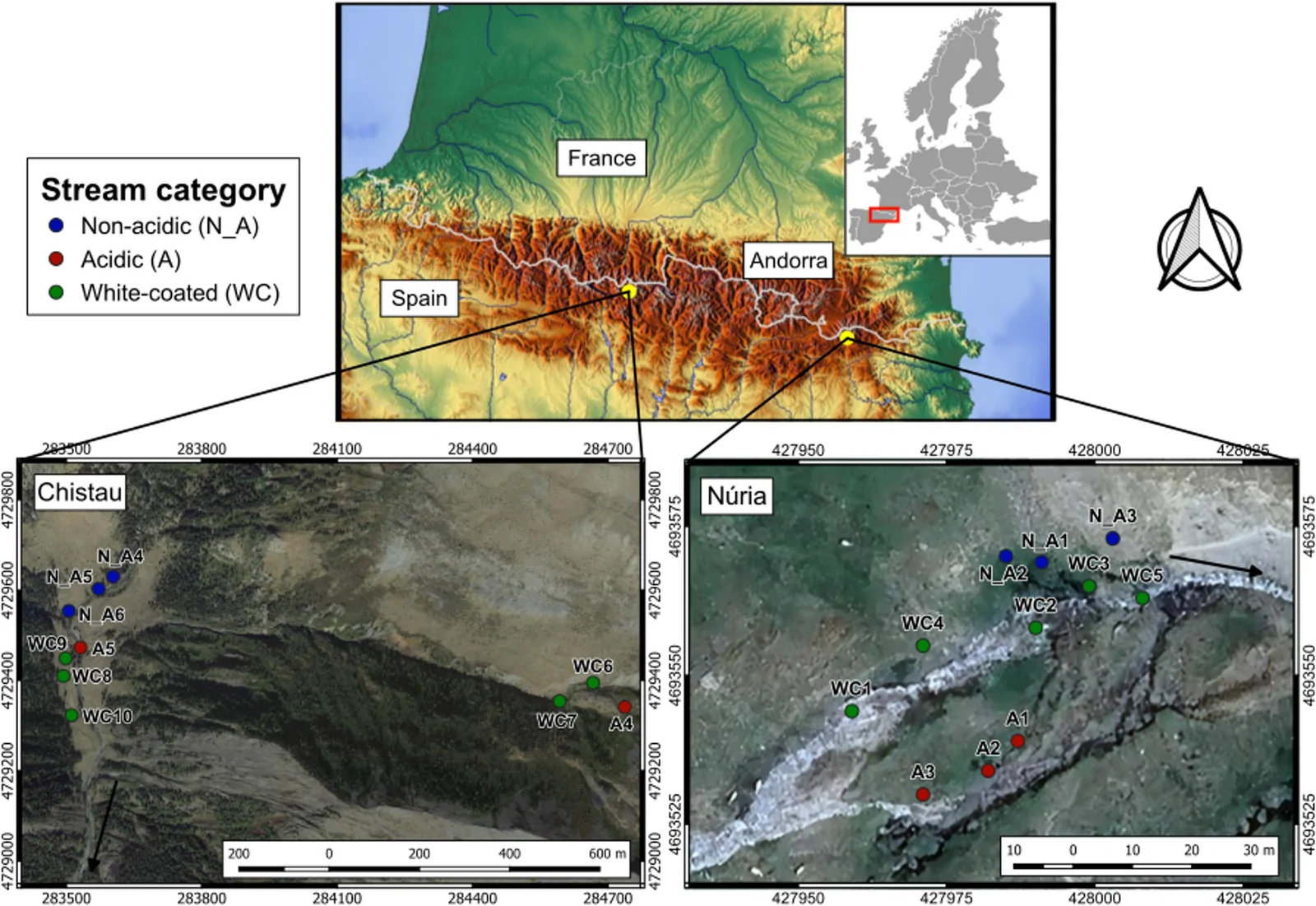
Geographical localization of the non-acidic (in blue), acidic (in red) and white-coated (in green) sampling sites in the Eastern and Central Pyrenees (Núria and Chistau, respectively). Black arrows indicate the direction of the water flow. Some sampling sites are away from the main stem as they were collected from nearby water sources

We sampled two Pyrenean regions: the Valley of Núria in the Eastern Pyrenees, within the “Capçaleres del Ter i Freser” Natural Park, and the Valley of Chistau in Central Pyrenees within the “Posets-Maladeta” Natural Park (Fig. 1). In Núria, the sampling was performed in the Coma de l’Embut stream (2300–2400 m a.s.l.), while in Chistau, sampling streams included the Bachimala Ravine (2100–2150 m a.s.l.) and the Cinqueta de la Pez River (1800–1900 m a.s.l.). Mean air annual temperature is 2.45 ± 0.13 °C in Núria and 4.95 ± 0.86 °C in Chistau, while annual precipitation averages 1247.18 ± 2.81 mm and 1495.85 ± 74.57 mm, respectively [19]. In Núria, the lithology is dominated by schists and slates, which are partially covered by rocky periglacial deposits produced by mass wasting processes of different natures. In Chistau, the lithology is dominated by slates and millimetric alternations of slates and quartzite, with hillside detrital deposits present in certain areas [20]. In addition, pyrite and arsenopyrite veins are present in the geological bedrock [21]. The vegetation is typical from the Pyrenean alpine and subalpine belt [22].

In Núria, neutral sampling sites receive groundwater discharged from the northern mountain slope. Meanwhile, acidic sites are supplied by groundwater from the southern slope, where sulfur-bearing materials are affected by an arrested translational slide, a type of rock slope failure that enhances water-rock interaction [23]. In Chistau, acidic waters come from the tributary (Bachimala Ravine), which discharges in the neutral main water course (Cinqueta de la Pez; Fig. 1).

In each region, we sampled epilithic biofilm of three stream categories: non-acidic, acidic, and confluences exhibiting a white-coated streambed constituted of metal precipitates. To that, the light and main water flow exposed surfaces (avoiding laterals) of randomly selected 3–4 cobble-size stones per site were brushed with soft toothbrushes, which provided sufficient biofilm biomass for robust community characterization. To avoid contamination between samples, each one was brushed with different toothbrushes. Biofilm slurries were split half frozen in a sterile Falcon tube at −21 °C for metabarcoding analysis, half preserved in ethanol 70% for diatom analysis. We sampled 11 river sites in Núria, and 10 in Chistau in late November-early December 2018 (Fig. 1, Supplementary Table 1). In the field, we measured stream water physicochemical variables with Hanna handheld meters with temperature compensation to 25 °C (stream water temperature and conductivity were measured with model HI98192 and pH with model HI98190).

**Table 1:** Taxa richness (D^0^) and evenness (EF0,1) of bacteria, eukaryotes and diatoms for both regions and stream type

Biodiversity Index	Region	Stream category	Bacteria			Eukaryotes			Diatoms		
			Mean	SD	*n*	Mean	SD	*n*	Mean	SD	*n*
Richness	Núria	Non-acidic	438	56	3	110	17	3	29	6	2
		Acidic	156	71	3	76	32	3	25	5	2
		White-coated	163	86	5	87	76	5	28	1	2
	Chistau	Non-acidic	378	8	3	76	25	3	13	-	1
		Acidic	214	57	2	123	48	2	11	-	1
		White-coated	211	39	5	133	29	5	25	5	4
Evenness	Núria	Non-acidic	0.62	0.05	3	0.22	0.10	3	0.47	0.07	2
		Acidic	0.39	0.04	3	0.29	0.19	3	0.33	0.10	2
		White-coated	0.35	0.11	5	0.20	0.11	5	0.39	0.11	2
	Chistau	Non-acidic	0.51	0.05	3	0.30	0.03	3	0.38	-	1
		Acidic	0.33	0.13	2	0.31	0.07	2	0.21	-	1
		White-coated	0.29	0.09	5	0.30	0.04	5	0.16	0.01	4

## Metabarcoding Analyses of Bacterial and Eukaryotic Communities

Samples were sent to AllGenetics & Biology SL (A Coruña, Spain). DNA was extracted using the DNeasy PowerSoil DNA isolation kit. To perform the PCRs, the primers Bakt 515 F (5’ GTG CCA GCM GCC GCG GTA A 3’) and Bakt 926R (5’CCG YCA ATT YMT TTR AGT TT 3’) were used for bacteria [24], while the primers TAReuk454FWD1 (5’ CCA GCA SCY GCG GTA ATT CC 3’) and TAReukREV3 (5’ ACT TTC GTT CTT GAT YRA 3’) were used for eukaryotes [25]. Samples were sequenced on Illumina MiSeq PE300, and sequences were trimmed and filtered implementing the DADA2 workflow in QIIME2 [26]. The taxonomy was assigned to ASVs (Amplicon Sequence Variant) using a pretrained classifier of the SILVA reference database for bacteria and the VSEARCH approach for eukaryotes, both implemented in QIIME2 [27, 28]. Afterwards, the ASVs with a frequency below 0.05% in each sample were removed to avoid mistagging. Finally, ASV assigned to chloroplasts have been deleted manually from the bacterial database.

### Sample Preparation and Identification of Diatom Communities

Samples were treated with 33% H_2_O_2_ and incubated in a hot bath at 60–70 °C for 24–48 h to remove organic matter. Subsequently, 1 M HCl was added to halt the reaction and eliminate potential carbonate interferences. The resulting supernatant of H_2_O_2_ and HCl was discarded, and the samples were washed three times with distilled water to remove residual reagents. A 24-hour interval was allowed between each washing step to enable sample precipitation and minimize material loss during supernatant removal [29]. After the final removal of distilled water supernatant, samples were placed in a slide using Naphrax (R.I: =1.7) for their observation in an optical microscope (Axioscope Zeiss with double immersion and objective 100x Apochromat 1.4 numeric aperture). A minimum of 350 diatoms were identified based on general diatom floras from Europe used in previous diatom studies [30] but the basionym currently accepted was used (http://www.algabase.org*).*

#### Data Analysis

Relationships among environmental variables (pH, temperature, and electrical conductivity) were tested with Spearman correlations. Alpha diversity of biofilm communities was estimated by calculating Hill numbers for richness (D^0^; Eq. 1) and the exponential of Shannon (D^1^; Eq. 2;) using the iNEXT package (version 3.0.1; [31]). In addition, evenness (EF_0,1_; Eq. 3) was calculated from previous Hill numbers [32]. Prior to these calculations, rarefaction curves were performed for standardizing by sampling effort (5,000 sequences for bacteria, 20,000 sequences for eukaryotes and 398 individuals for diatoms; Supplementary Fig. 1a, b, c).

1 $$\:{D}^{0}={\left({\sum\:}_{i=1}^{S}{p}_{i}^{0}\right)}^{1/(1-0)}$$

2 $$D^1=e^H=exp\left(-{\textstyle\sum_{i=1}^S}P_i\cdot Inpi\right)$$

3 $$\:{EF}_{\mathrm{0,1}}=\:\frac{{D}^{1}}{{D}^{0}}$$

Differences in the diversity indices across regions and stream categories were tested with Type II or Type III two-way ANOVA based on the significance of factor interaction to account for unbalanced data [33]. For eukaryotes, the exponential of Shannon was transformed with natural logarithm for accomplishing normality and homoscedasticity statistical assumptions. Differences in EF_0,1_ for diatoms were tested performing a Welch test, since it did not meet the homoscedasticity assumption. The number of ASVs and diatom species exclusive to or shared between stream categories and regions were visualized the with Venn diagrams using the R package *BiodiversityR* (version 2.16.1 [34]),.

Non-metric multidimensional scaling (NMDS) analysis based on Bray-Curtis distances was used to compare relative abundances of bacteria, eukaryote and diatom communities across regions and stream categories. Classes, phyla, and species of bacteria, eukaryotes, and diatoms were fitted to each NMDS to identify significant taxa (*P* < 0.05) for each region and stream category using the *envfit* function of the vegan package (version 2.6–8.6; [35]). The significance of region, stream category, and their interaction was then evaluated with PERMANOVA, also implemented in the vegan package.

Indicative microbial taxa were determined for acidic and white-coated streams using the Indicator Value (IndVal) analysis with the *indicspecies* library (version 1.7.14; [36]). This method combines the species relative abundance with its relative frequency of occurrence to statistically determine species associated with site categories.

Co-occurrence networks of each stream category were analysed for both Núria and Chistau regions separately, using the sparse compositional correlations method [37] with the *sparcc* function included in the R package *SpiecEasi* (version 1.1.3; [38]) and correlations with absolute coefficients of >0.6 were reported. Co-occurrence network graphics and their linkage densities (D) were calculated using the R package *igraph* (version 2.1.1; [39]). The limitation of sample number prevented us from conducting co-occurrence analyses in acidic streams in Chistau, as well as for all diatom communities. All the statistical analyses were carried out with RStudio (version 9.0.375 [40]),.

## Results

Streams in both regions had a similar range of pH values, from 4.0 to 7.2, and water temperatures ranging from 0.4 to 3.3 °C (Supplementary Table 1). Contrastingly, electrical conductivity was higher in Núria (130 to 541 µs/cm) than in Chistau (9 to 100 µs/cm; Supplementary Table 1). Lowest pH and highest electrical conductivities were associated with the coldest water temperatures (Supplementary Fig. 2).

**Fig. 2:**
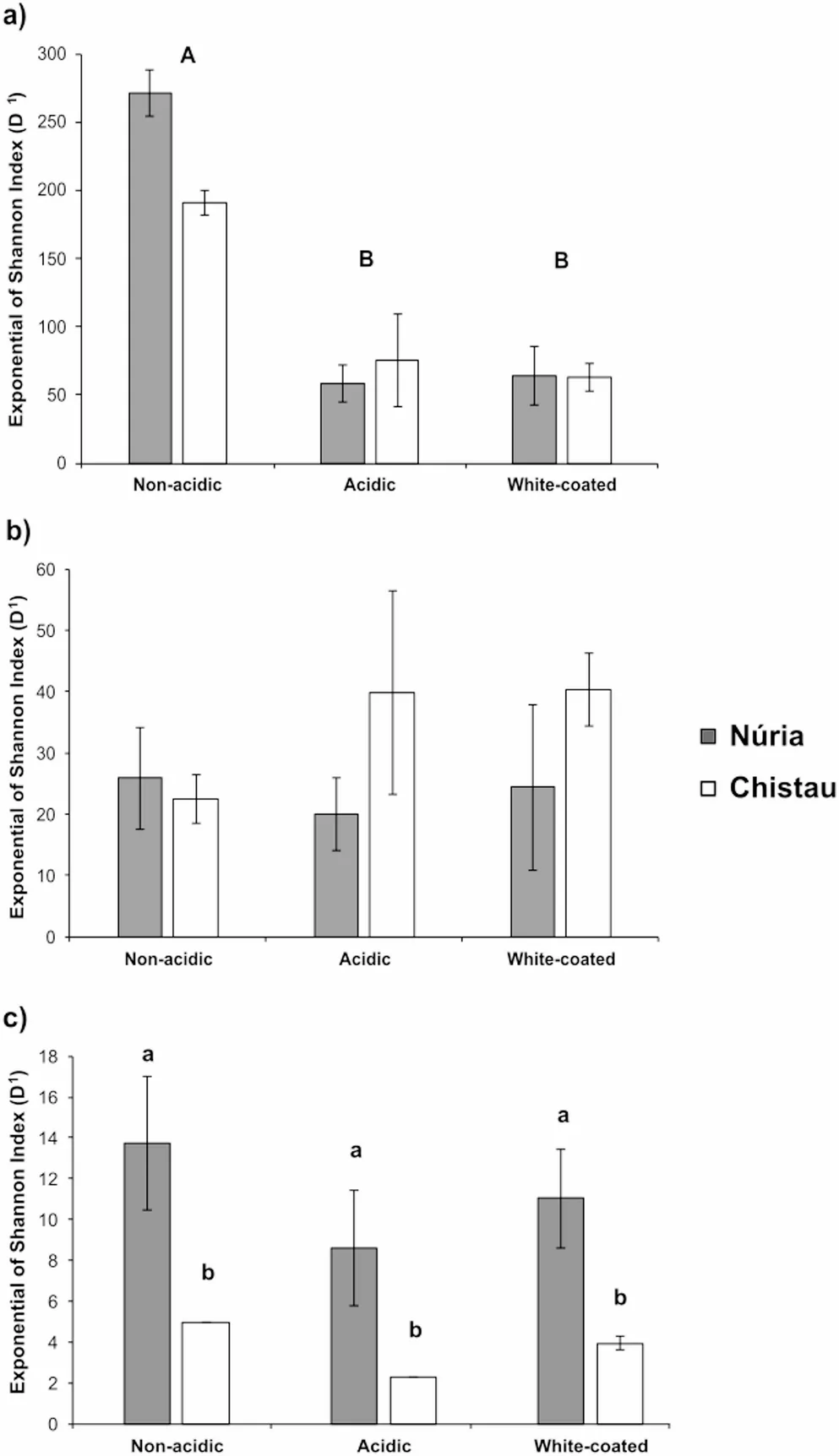
Shannon´s diversity indices (H’) of bacteria (**a**), eukaryotes (**b**) and diatoms (**c**) for stream types (Non-acidic, Acidic, and White-coated) and regions (Núria and Chistau). Mean values ± standard errors are shown, except for diatoms in “Non-acidic” and “Acidic” sites from Chistau (number of samples in Table 2). Capital letters stand for significant differences among stream categories (ANOVA test, *p* = 0.002), and lowercase letters for differences between regions (ANOVA test, *p* < 0.001)

Alpha diversity indices (i.e. D^0^, D^1^, and EF_0,1_) were consistently higher in non-acidic compared to acidic and white-coated for bacterial communities (ANOVA, *P* < 0.001, emmeans tests, *P* < 0.001; Fig. 2; Table 1). Venn diagrams confirm this trend, showing a greater number of exclusive bacterial ASVs to non-acidic streams (893 in Núria and 610 in Chistau) compared to those exclusive to acidic and white-coated streams (from 207 to 391 ASVs considering both regions; Supplementary Fig. 3a).

**Fig. 3:**
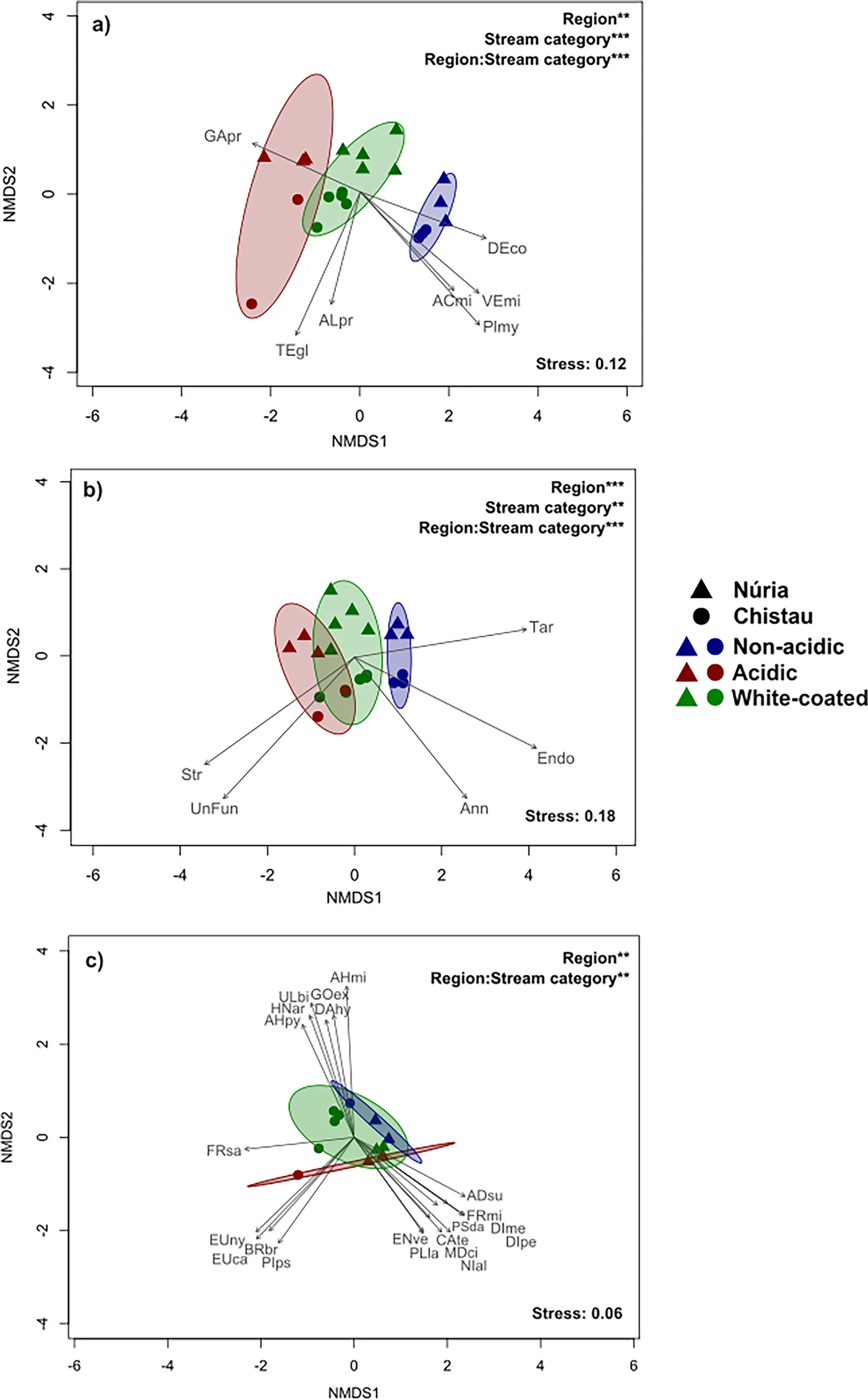
Non-metric multidimensional scaling (NMDS) plots illustrating the similarity of sampling sites for bacteria (**a**), eukaryotes (**b**), and diatoms (**c**). Stream categories are distinguished by colours, and regions are represented by shapes. Ellipses represent 85% confidence regions. Arrows indicate significant taxonomic associations (classes for a and phyla for b, α = 0.05 and species for c, α = 0.01, 9999 permutations). Complete results of the PERMANOVA tests are shown in Supplementary Table 2, but p-value significance levels are indicated in the graphics with the following code: ** ≤0.01, *** ≤0.001. Refer to Supplementary Table 3 for taxon codes details

**Table 2:** Bacteria and eukaryote indicator taxa following the Indicator Value Method for acidic and white-coated rivers (p-value < 0.05, 9999 perm.). Numbers in brackets refer to the bibliography from the text.

Type of organism	Stream category	IndVal		*p*-value	Taxon (Class, Order, Family, Genus)	Potential traits of interest
Bacteria	Acidic	100.00		< 0.001	*Acidiimicrobia*	Acidophilic, iron oxidizer [52]
		98.70		< 0.001	*Rhodanobacter*	Acidophilic, denitrifier [50]
		97.60		< 0.001	*Chitinophagaceae*	-
		94.80		< 0.001	*Rhodovastum*	Acidophilic [51],
		94.70		< 0.001	*Granulicella*	Acidophilic, psychrotolerant [53]
		89.40		< 0.001	*Rhizorhapis Singulisphaera*,* Pedosphaeraceae*	- Moderate acidophilic [54] -
		88.10		0.002	*Rhizorhapis*	-
		87.10		0.002	*Holophaga*	-
		85.00		0.002	*Nitrosomonadaceae*	Nitrifier [55]
		83.70		0.007	*Rhodovastum*	Acidophilic [51]
		80.80		0.007	*Bradyrhizobium*	-
		77.50		0.008	*Holophaga*	-
		77.50		0.007	*Xanthomonadaceae*	-
		77.50		0.006	*Huanghella*, *Gemmatimonadaceae*,* Armatimonadales*,* Novosphingobium*,* Pseudanabaena*,* Sphingomonadaceae*	- - - - -
	White-coated		98.80	< 0.001	*Pseudanabaena*	-
		94.30		< 0.001	*Microscillaceae*	-
		93.30		0.003	*Methylotenera*	-
		91.90		< 0.001	*Shingobacteriales*	-
		86.10		0.008	*Chamaesiphon*	-
		85.60		0.004	*Fimbriiglobus*	Moderate acidophilic, psychrotolerant [68]
		83.00		0.013	*Schlesneria*	Moderate acidophilic [67]
		82.00		0.012	*Cyanophyceae*	N fixer [66]
		81.90		0.015	*Parasediminibacterium*	-
		77.70		0.049	*Deinococcus*	Extremophilic [69]
		77.50		0.014	*Elusimicrobia*	-
		70.70		0.03	*Anaerolineae*	-
Eukaryote	Acidic	98.80		< 0.001	*Chrysophyceae*	-
		93.32		< 0.001	*Helotiaceae*	-
		77.26		0.0120	*Chrysocapsa paludosa*	-
	White-coated	81.36		0.0478	*Chrysophyceae*	-

In contrast, diversity indices for eukaryote communities did not differ among stream categories nor regions (Fig. 2; Table 1) though white-coated streams showed more unique eukaryotic ASVs (183 in Núria and 203 in Chistau) compared to non-acidic and acidic streams (from 58 to 177 ASVs in both regions; Supplementary Fig. 3b). For diatoms, diversity indices differed mainly between regions (Fig. 2c; Table 1), with higher values in Núria than in Chistau of D^0^ (ANOVA, *P* = 0.022), D^1^ (Welch test, *P* = 0.005) and EF_0,1_ (Welch test, *P* = 0.004). Diatom richness and evenness were also significantly higher in Núria than in Chistau, respectively (Table 1). Additionally, while not statistically significant, diatom communities tended to exhibit a slightly higher D^1^ in non-acidic streams (from 5.0 to 17.0) than in acidic and white-coated (from 2.3 to 11.4 and 3.0 to 13.5 respectively).

Bacterial community composition varied across both stream category and region (Fig. 3a and Supplementary Table 2). The first axis of NMDS organized communities by stream category, with acidic on the left and non-acidic on the right, while white-coated were in between and closer to the acidic ones. The second axis distinguished Núria (top) from Chistau (bottom) communities, with greater similarity among non-acidic streams than among ARD-affected ones. Communities from acidic streams were characterized by *Gammaproteobacteria*, and white-coated ones from Chistau by *Terriglobia* and *Alphaproteobacteria*. Meanwhile, communities in non-acidic streams were characterized by *Acidomoicrobia*,* Deinococci*,* Planctomycetia* and *Verrucomicrobia* classes.

**Fig. 4:**
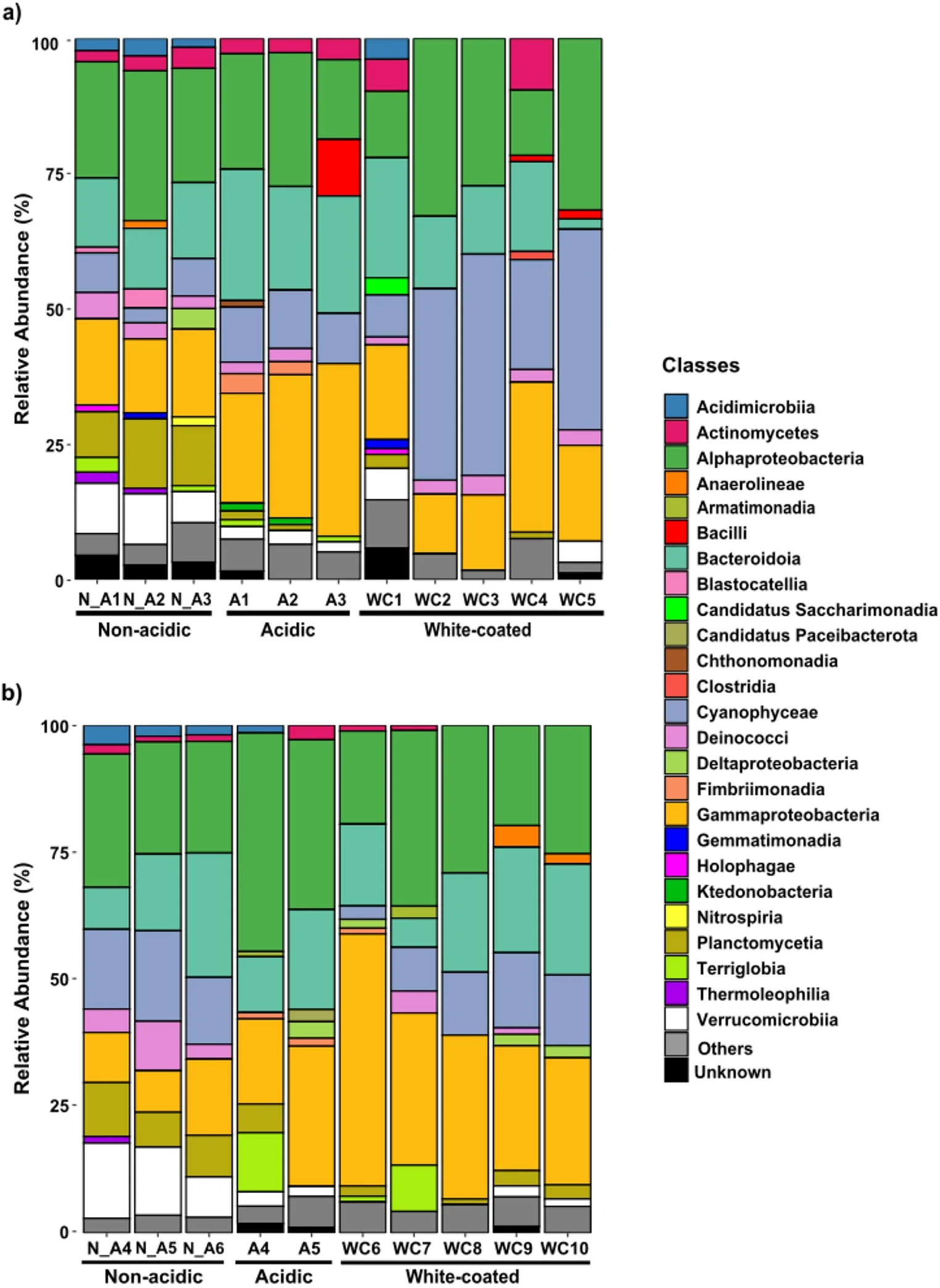
Relative abundance of main bacteria classes for: (**a**) Núria and (**b**) Chistau (except for “Candidatus Paceibacterota” and “Candidatus Saccharimonadia, which are phyla). The category “Others” stands for those taxa that have < 1% of relative abundance in each sample

The bacterial community composition in terms of class abundances is summarized in Fig. 4. In Núria, *Gammaproteobacteria* (10–30%) were more abundant in acidic and white-coated steams than in non-acidic ones (10–15%). Meanwhile, *Cyanophyceae* (10–40%) were more abundant in white-coated streams than in acidic and non-acidic streams (5–10%). In Chistau, *Gammaproteobacteria* were also more abundant (20–50%) in ARD-affected streams, as well as *Alphaproteobacteria* (20–35%) compared to non-acidic streams (10–20%). Also, *Cyanophyceae* were absent in acidic streams, while there were present in non-acidic and white-coated streams (5–20%; Fig. 4a and b).

Similarly, eukaryotic community composition also exhibited significant differences according to stream category (Fig. 3b; Supplementary Table 2). White-coated stream communities remained intermediate and closer to acidic ones, though differences were less evident to those observed for bacteria. Acidic communities, particularly in Chistau, were characterized by *Streptophyta* and *Unknown fungi*, while non-acidic communities in Núria were characterized by *Tardigrada*, while *Endomyxa* and *Annelida* were more associated with Chistau communities.

**Fig. 5:**
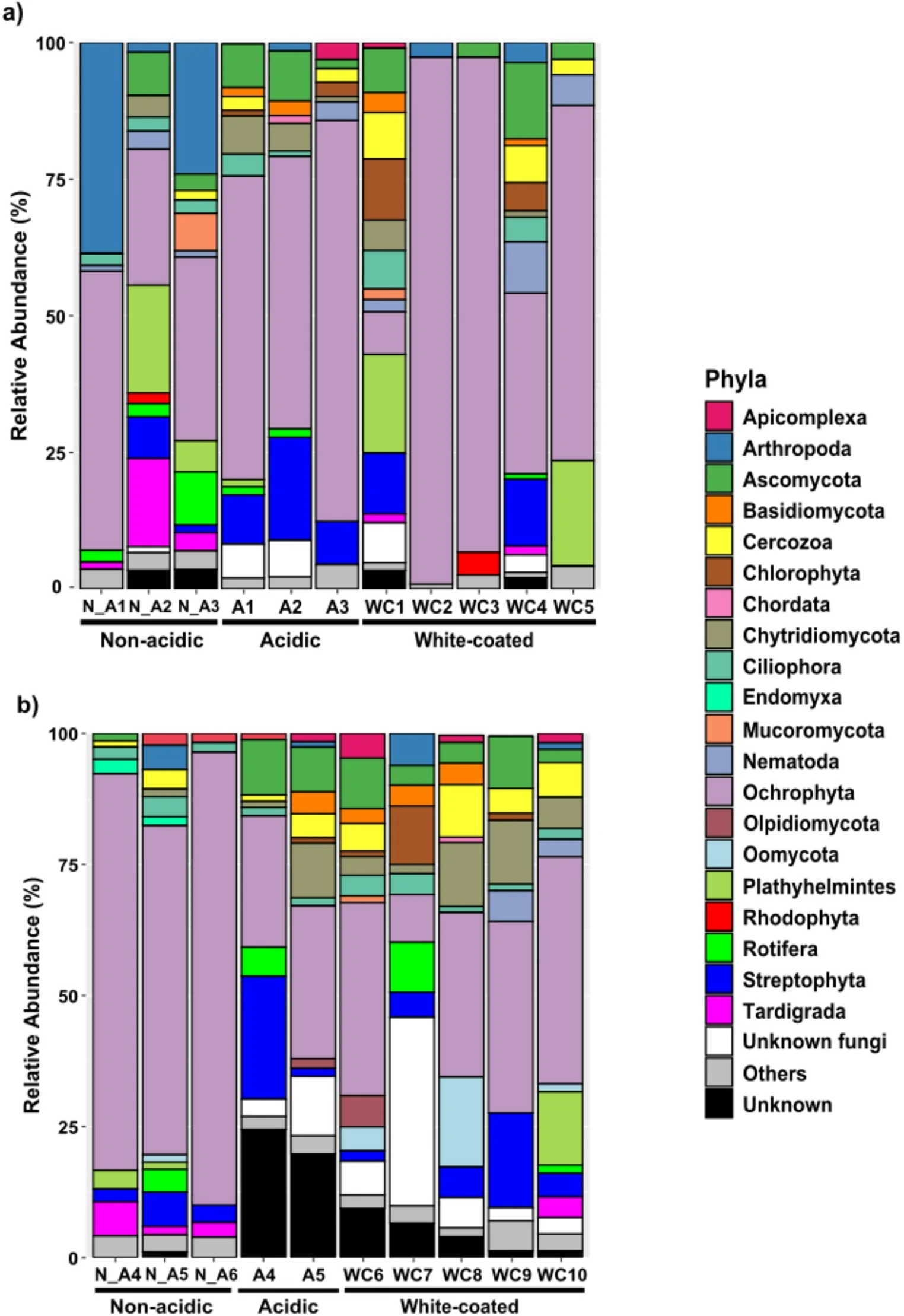
Relative abundance of the main eukaryotic phyla for Nuria (**a**) and Chistau (**b**). The category “Others” stands for those taxa that have < 1% of relative abundance in each sample

The eukaryote community composition by relative phyla abundances is summarized in Fig. 5. The most abundant phylum across regions was *Ochrophyta* (10–95%). Fungal groups (including *Ascomycota*,* Basidiomycota*,* Chytridiomycota*,* Mucoromycota*,* Olpdiomycota* and *Unkown fungi*) were more abundant in acidic and white coated streams (5–25%) than in non-acidic ones (1–10%). *Streptophyta* exhibited higher abundances in ARD-affected streams (10–20% in Núria and 2–20% in Chistau) than in non-acidic streams (1–10% and 1–5% respectively). Interestingly, *Chlorophyta* did only appear in samples from acidic and white-coated streams in both regions (Fig. 5a and b). Finally, co-occurrence networks revealed that both, bacterial and eukaryote communities, were less connected (exhibited lower density networks) in white-coated than in acidic and non-acidic streams (Supplementary Fig. 4).

Diatom communities differed significantly between regions, while the interaction between region and stream category was also significant (Fig. 3c and Supplementary Table 2). The horizontal axis of the NMDS plot separated Núria (right) from Chistau (left) communities, while vertical axis tended to separate communities according to stream category. In Chistau, acidic samples were characterized by the *Eunotia* genus, *Frustulia saxonica* was related to white-coated communities, while non-acidic communities were characterized by the *Achnanthidium* genus. In Núria, *Encyonema ventricosum* and *Planothidium lanceolatum* were associated with acidic communities, while *Adlafia suchlandtii* appeared to be more related to white-coated communities (Fig. 3c; Supplementary Fig. 3).

**Fig. 6:**
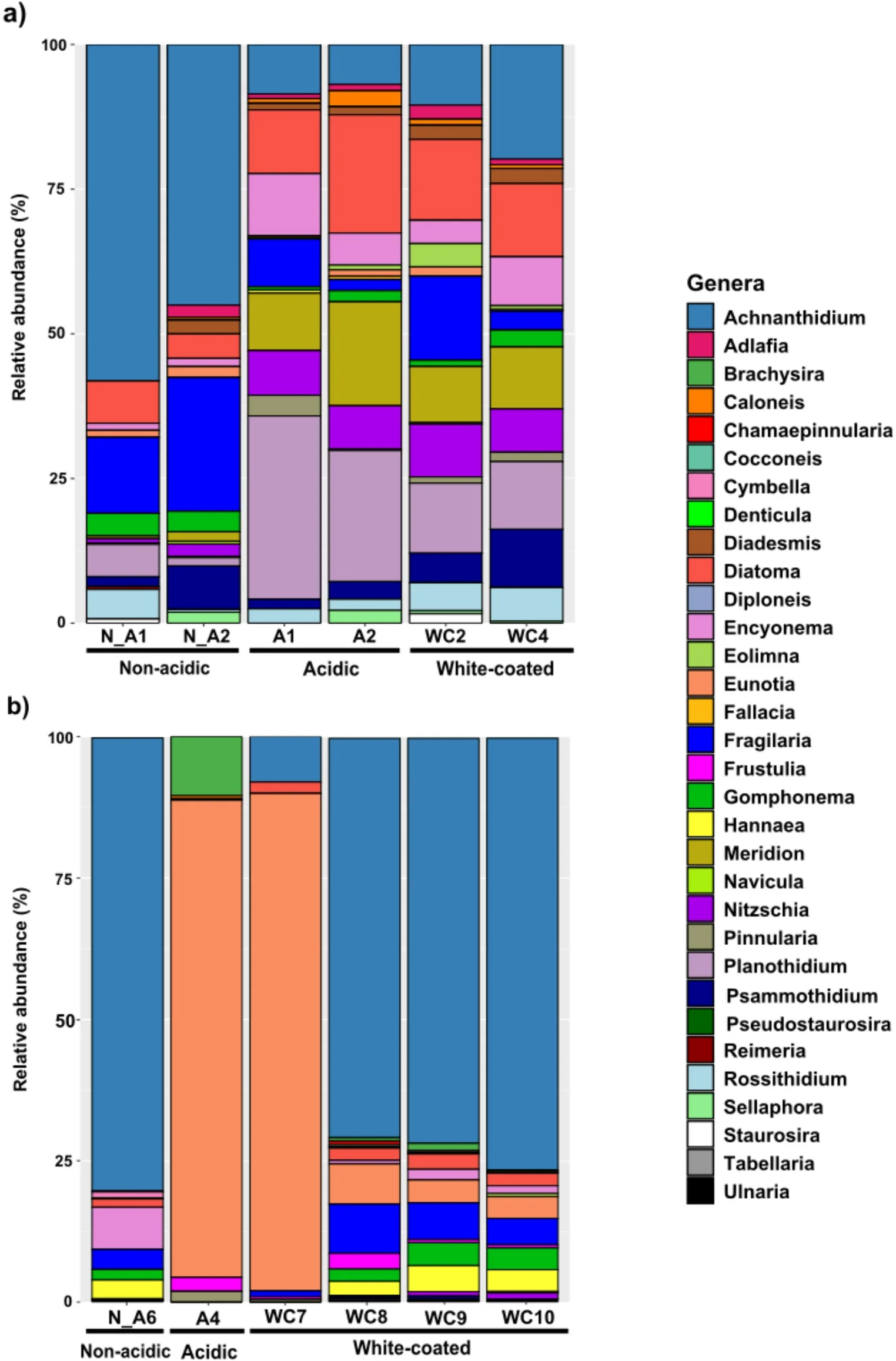
Relative abundance of diatom genera for Núria (**a**) and Chistau (**b**)

Diatom community composition in terms of genus relative abundances is summarized in Fig. 6. Relative abundances of *Achnanthidium* (45–60%) and *Fragilaria* (15–25%) dominated in communities of non-acidic streams from Núria, while *Planothidium*, *Meridion*, *Diatoma* (each 10–20%) and *Nitzschia* (5–10%) predominated in ARD-affected streams (Fig. 6a). In Chistau, *Achnanthidium* was also dominant in non-acidic and in three of the four white-coated samples (up to 80%), while *Eunotia* dominated in acidic and one of the white-coated samples (85–90%; Fig. 6b).

The indicator taxa of acidic sites included ASVs from *Acidiimicrobia*, followed by *Rhodanobacter*,* Chitinophagaceae*,* Rhodovastum*, and *Granulicella* (IndVal > 94, *p* < 0.001; Table 2). Also, *Rhodovastum*,* Holophaga* and *Rhizorhapis* genera were assigned not one, but two indicator ASVs each. For white-coated streams, an ASV from the genus *Pseudanabaena* had the highest IndVal, followed by ASVs of *Microscillaceae*,* Methylotenera*, and *Shingobacteriales* groups (IndVal > 90, *P* ≤ 0.003; Table 2).

For eukaryotes, ASVs of the *Chrysophyceae* family had the highest IndVal both for acidic and white-coated streams (Table 2). We also found the *Helotiaceae* family and *Chrysocapsa paludosa* to be indicators of acidic streams (IndVal > 77.26, *p* ≤ 0.0120; Table 2). For diatom species, only *Fallacia vitrea*,* Fragilaria capucina*,* Gomphonema hebridense*,* Pinnularia pseudogibba* approached the significance threshold in acidic streams (IndVal = 66.59, *p* = 0.091; Supplementary Table 4).

## Discussion

Our study provides insights into the effects of ARD on mountain freshwater ecosystems by comparing ARD-affected and non-affected waters in two Pyrenean valleys. Consistent with previous research, the inverse relationship between pH and conductivity suggests that conductivity increase is driven by the sulphate salts dissolution and the pH decrease by the sulfuric acid produced [41]. The positive correlation between pH and water temperature is less certain due to the low variability in temperature (0.4–3.3 °C), but it still suggests that ARD tends to originate in the coldest headwaters [7]. These hydrochemical conditions influenced microbial biofilms, with bacterial communities displaying the highest sensitivity to ARD compared to eukaryotic groups. Also, communities from ARD-affected streams differed from those in non-acidic ones in the same valleys, suggesting that natural ARD may promote regional biodiversity by increasing regional habitat heterogeneity in these remote and cold natural environments.

Our results clearly showed that ARD decreased bacterial diversity, as previously observed in streams affected by acid drainages [42]. In contrast, ARD did not decrease eukaryotic diversity overall, neither decreased the diatom community diversity. This differential response could be because eukaryotic cells are structurally and functionally more complex than bacterial cells [43], facilitating their adaptation to the changing environmental conditions and diminishing the environmental filtering effect at the community level [44]. However, a decrease in eukaryotic diversity was expected as it has been shown in streams affected by human induced acid mine drainages, although these commonly reach lower pH than those attained in this study [11]. Similarly, diatoms are described as good indicators of acidification and commonly exhibit a decrease in alpha diversity with decreasing pH [45]. The lack of a significant effect in this study could be because most of our sites had pH values around 5, while the major diversity decrease for diatoms is expected at pH values around 4.5–3.5 [46]. Overall, although our pH range was not very large, the observed effect of ARD on microbial biofilm diversity, especially eukaryotic, was less strong than expected. This suggests that this process occurring in alpine pristine rivers might favour the development of communities adapted to these natural acidic environments, which could be a different effect to those generated from mining activities from which most studies are referred to [15, 17].

The community composition of the three studied microbial groups clearly differed between the non-acidic and the acidic stream conditions. Interestingly, communities in non-acidic streams were more similar across regions than in ARD-affected ones. This is relevant, because normally the most important factor to consider on microbial communities is the region and its climate above other local variables [47]. However, in this case acidity is nearly as significant as the region. This suggests that the hydrochemical difference acts as a filter selection, causing a significant change in community composition between non-acidic and acidic streams for the three studied groups.

In the case of bacteria, two of the most abundant orders found in acidic stream biofilms, *Alphaproteobacteria* and *Gammaproteobacteria*, are known to include both iron- and sulphide-oxidizing bacteria [48, 49]. This is also supported by the indicator taxa analyses since some indicator ASVs of acidic streams were assigned to acidophilic, iron oxidizing, and psychrotolerant bacteria [50–54]. Interestingly, other indicator taxa of acidic streams are related to environmental nitrification and denitrification [50, 55]. Additionally, similar density values in acidic and non-acidic microbial networks suggest comparable stability in bacterial communities. Since stressed communities by environmental perturbations typically show lower density values, this may indicate that acidic conditions may have been stable enough over time, thereby providing sufficient opportunity for microbial adaptation [56, 57].

For eukaryotes, acidic and white-coated streams favoured some photosynthetic groups (*Streptophyta* and *Chlorophya*), as well as fungi. Photosynthetic organisms rely on nutrients and metals such as iron for photosynthesis. Acidic rivers with higher solute concentrations may support these organisms in colder environments, where metabolic rates are typically slowed [58, 59]. This effect could be reinforced by the presence of nitrogen-fixing bacteria previously stated, since it would enhance benthic nutrient availability within biofilm, too [60]. For fungi, their increase may be due to their tolerance to different pH levels and their ability to sequester metals through cell walls or extracellular polysaccharides [61, 62]. However, none of eukaryote indicator taxa was linked to these groups but related to *Chrysophyceae*, which have been previously used for studying natural acidification of alpine pristine freshwater ecosystems in other studies [63]. In the case of diatom communities, they exhibited only minor differences among stream categories, likely due to overriding influence of regional factors on diversity and community structure, as well as limited observations. Nevertheless, clear compositional shifts are evident despite this strong regional effect, with communities in non-acidic streams differing from those in non-acidic systems, and including genera typically associated with more acidic environments (i.e. *Eunotia*, *Nitzschia*) [46].

Similarly to bacteria, eukaryote co-occurrence networks from non-acidic streams had similar densities to the acidic ones, reinforcing the idea of these acidic environments being stable over time and allowing their communities to adapt properly. These results, together with the non-reduced eukaryotic diversity in the acidic streams discussed before, suggest that the microbial community response to natural ARD being different to acid mine drainages. Differences in origin and acidification rates likely underlie these different responses, supporting the view that mine drainages constitute a distinct perturbation than natural ARD [16–18].

Although diversity indexes were similar in acidic and white-coated streams, community composition in white-coated stream biofilms differed markedly. White-coated microbial communities were distinct, sharing taxa with both acidic and non-acidic streams. However, certain particularities emerged depending on the biological group considered. Bacteria classes which tolerate high concentrations of metals and can use metals in their metabolisms (i.e. *Alphaproteobacteria*) or sulphur-reducing taxa (i.e. *Terriglobia*) were favoured in these white-coated streams [48, 64]. Additionally, *Cyanophyceae* were more abundant in these streams, likely due to their considerable adaptability and versatility. This would be conferred by their distinctive cell structure and their ability to form filaments and colonies [65].

An indicator ASV in white-coated streams is also linked to *Cyanophyceae* group, recognized for their ability to fix atmospheric nitrogen [66]. However, there are other indicator taxa included into the *Cyanophyceae* group such as *Pseudanabaena*, *Microscillaceae* and *Chamaesiphon* that are not related to atmospheric nitrogen fixation. In addition, other indicator taxa were associated with moderate acidophilic, psycrotolerant and extremophilic bacteria [67–69].

The co-occurrence networks suggested that microbial communities in white-coated streams are the most unstable (i.e. with lowest resistance and resilience to perturbations), as indicated by lowest network density values. Whitish precipitates are formed by metastable aluminium hydroxides and hydroxy-sulphates, and seasonal hydrological fluctuations from different water sources drive pH variations, which in turn control mineral precipitation and white-coatings formation [7]. These periodic perturbations may increase environmental variability in these water-mixing areas, potentially hampering the development of stable microbial communities [56]. However, our study is based on one single point representing a snapshot in time, not including seasonal or interannual variability. Also, the limited and uneven number of diatom samples may have constrained interpretation, affecting spatial representativeness and statistical power. Given that sampling was conducted in winter, a detailed temporal analysis during summer, when microbial activity is typically higher, would be beneficial. That would allow the persistence assessment of these patterns and to better understand the dynamics and resilience of microbial communities under varying environmental conditions.

Above all the observed community diversity changes across stream categories, the regional factor was further determining community characteristics. All three studied biological groups showed differences in the community composition between the two studied valleys and lower diatom diversity in Chistau than in Núria. The greater similarity in closer communities could be related to geographic distance and easier dispersion as well as local environmental conditions when sharing the same watershed [47]. However, the shifts from non-acidic to acidic and white-coated streams in terms of community composition were occurring in parallel at the two valleys, strengthening the observed patterns. This is even more remarkable considering the differences in spatial scales between the sampling sites, much larger in Chistau (from hundreds of meters to a few km) than in Núria (only a few meters).

Natural ARD in alpine watersheds play a key role in modifying regional biodiversity creating a mosaic of distinct habitats in small geographical distances. Overall, ARD effects on alpine microbial biofilm communities may be a balance between stress effects and tolerance/adaptive strategies, with also different effects at the directly ARD-affected streams (acidic) to those where metal precipitation occurs in confluences (white-coated).

## Supplementary Information

Below is the link to the electronic supplementary material.

Supplementary Material 1 (DOCX. 16.4 MB)

## Data Availability

ASVs ID data, taxonomical assignations and absolute abundances for prokaryotic 16 S rRNA and eukaryotic 18 S rRNA, as well as diatom absolute abundances that support the findings of this study have been deposited in the Catalan Open Research Area with the following DOI: https://doi.org/10.34810/data2501.
