# Supplementary material for: Effects of Acid Rock Drainage on Microbial Communities in Alpine Streams of the Pyrenees
Source: Microb Ecol. 2025 Dec 5;89:17. doi: 10.1007/s00248-025-02667-1 (PMC12790537; doi:10.1007/s00248-025-02667-1)
Supplement: Supplementary file 1 [file 248_2025_2667_MOESM1_ESM.docx]

**Supplementary Tables and Figures**

# Effects of acid rock drainage on microbial communities in alpine streams of the Pyrenees

*Microbial Ecology*

José Luis Guijosa-Ortega*^1^, Anna M. Romaní^1^, Oriol Grau^2^, Sergi Pla-Rabes^3,4^Olga Margalef ^3,5^, José Gabriel Salminci^6^, Mario Zarroca^6^, Ada Pastor^1^

^1^GRECO, Institute of Aquatic Ecology, University of Girona, Girona, Spain

^2^Parc Natural de l’Alt Pirineu, Llavorsí, Lleida 25595, Spain

^3^BABVE, Autonomous University of Barcelona, Cerdanyola del Vallès, Barcelona, Spain

^4^CREAF, Cerdanyola del Vallès, Barcelona, Spain

^5^RISKNAT Research Group, Department of Earth and Ocean Dynamics, University of Barcelona, Spain

^6^External Geodynamics and Hydrogeology Group, Autonomous University of Barcelona, Cerdanyola del Vallès, Barcelona, Spain

*Contact author: [joseluis.guijosa@udg.edu](mailto:joseluis.guijosa@udg.edu)

**Supplementary Table 1** Universal Transverse Mercator geographical coordinates (UTM, 31N) and physicochemical characterization of the sampling sites

| **Region** | **Code** | **Stream type** | **UTM (X)** | **UTM**  **(Y)** | **pH** | **Conductivity**  **(µs/cm)** | **Temperature**  **(ºC)** |
| --- | --- | --- | --- | --- | --- | --- | --- |
| Núria | N_A1 | Non-acidic | 427991 | 4693569 | 6.7 | 130 | 3.8 |
|  | N_A2 | Non-acidic | 427985 | 4693570 | 7.1 | 151 | 2.7 |
|  | N_A3 | Non-acidic | 428003 | 4693573 | 7.0 | 114 | 2.7 |
|  | A1 | Acidic | 427987 | 4693539 | 5.4 | 521 | 0.8 |
|  | A2 | Acidic | 427982 | 4693534 | 5.1 | 514 | 1.1 |
|  | A3 | Acidic | 427971 | 4693530 | 5.3 | 541 | 1.1 |
|  | WC1 | White-coated | 427959 | 4693544 | 6.4 | 297 | 0.6 |
|  | WC2 | White-coated | 427990 | 4693558 | 6.1 | 327 | 1.2 |
|  | WC3 | White-coated | 427999 | 4693565 | 6.5 | 276 | 0.8 |
|  | WC4 | White-coated | 427971 | 4693555 | 7.1 | 219 | 1.1 |
|  | WC5 | White-coated | 428008 | 469356 | 7.1 | 157 | 1.5 |
| Chistau | N_A4 | Non-acidic | 283603 | 4729630 | 6.6 | 53 | 3.2 |
|  | N_A5 | Non-acidic | 283571 | 4729603 | 7.1 | 20 | 3.2 |
|  | N_A6 | Non-acidic | 283505 | 4729554 | 7.4 | 9 | 3.3 |
|  | A4 | Acidic | 284743 | 4729347 | 4.0 | 77 | 0.4 |
|  | A5 | Acidic | 283531 | 4729473 | 5.0 | 100 | 0.1 |
|  | WC6 | White-coated | 284667 | 4729395 | 6.5 | 73 | 0.8 |
|  | WC7 | White-coated | 284593 | 4729354 | 6.2 | 70 | 1.1 |
|  | WC8 | White-coated | 283498 | 4729449 | 6.6 | 80 | 2.7 |
|  | WC9 | White-coated | 283493 | 4729410 | 6.8 | 80 | 2.8 |
|  | WC10 | White-coated | 283511 | 4729323 | 7.2 | 80 | 2.8 |

**Supplementary Figure 1** Rarefaccion curves for: a) Bacteria, b) Eukaryotes, and c) Diatoms. a) and b) are edited from rarefaction curves provided by AllGenetics&Biology SL. Continuous lines indicate real observations, while discontinuous lines indicate extrapolations made by iNEXT

**Supplementary Figure 2** Spearman correlations (Rho) between: a) pH-Temperature, b) pH-Conductivity and c) Conductivity-Temperature for both regions.

**Supplementary Figure 3** Venn diagrams of prokaryote (a), eukaryote (b) and diatom (c) communities, showing ASVs or diatom species specific or shared among regions (Núria and Chistau) and stream types (Non-acidic, Acidic and White-coated)

**Supplementary Table 2** Statistical results of PERMANOVA analysis performed for NMDS graphics in figure 3.

| ***Source of variation*** | | ***Bacteria*** | | | |  | ***Eukaryotes*** | | | | | |  | ***Diatoms*** | | | | | |
| --- | --- | --- | --- | --- | --- | --- | --- | --- | --- | --- | --- | --- | --- | --- | --- | --- | --- | --- | --- |
|  |  | ***R^2^*** | ***F*** | | ***p*** |  | ***R^2^*** | | ***F*** | | ***p*** | |  | ***R^2^*** | | | ***F*** | | ***p*** |
| Region | | 0.12 | 2.48 | | 0.006 |  | 0.16 | | 3.66 | | 0.001 | |  | 0.31 | | | 4.56 | | 0.010 |
| Stream category | | 0.29 | 3.69 | | 0.001 |  | 0.19 | | 2.11 | | 0.002 | |  | 0.29 | | | 1.79 | | 0.102 |
| Region:Stream Category | | 0.56 | 3.77 | | 0.001 |  | 0.48 | | 2.82 | | 0.001 | |  | 0.77 | | | 3.93 | | 0.007 |
|  |  | | |  | | | |  | |  | |  | | |  |  | |  |  |

**Supplementary table 3** Codes for bactertial classes, eukaryotic phyla and diatom species used in NMDS. Also, R^2^ and p-values from the envfit test in R are shown.

| ***Biological group*** | ***Taxonomical assignation*** | ***Codes*** | ***R^2^*** | ***P*** |
| --- | --- | --- | --- | --- |
| Bacteria | Acidimicrobiia | ACmi | 0.41 | 0.008 |
|  | Alphaproteobacteria | Alpr | 0.30 | 0.004 |
|  | Deinococci | DEco | 0.40 | 0.018 |
|  | Gammaproteobacteria | GApr | 0.31 | 0.040 |
|  | Planctomycetia | PLmy | 0.71 | <0.001 |
|  | Terriglobia | TEgl | 0.55 | 0.003 |
|  | Verrucomicrobiia | VEmi | 0.54 | <0.001 |
| Eukaryotes | Annelida | Ann | 0.27 | 0.046 |
|  | Endomyxa | Endo | 0.35 | 0.008 |
|  | Streptophyta | Str | 0.29 | 0.046 |
|  | Tardigrada | Tar | 0.26 | 0.043 |
|  | Unknown Fungi | UnFun | 0.32 | 0.010 |
| Diatoms | *Achnanthidium minutissimum* | AHmi | 0.91 | 0.001 |
|  | *Achnanthidium pyrenaicum* | AHpy | 0.61 | 0.013 |
|  | *Adlafia suchlandtii* | ADsu | 0.62 | 0.014 |
|  | *Caloneis tenuis* | CAte | 0.48 | 0.049 |
|  | *Diadesmis perpusilla* | DIpe | 0.73 | 0.004 |
|  | *Diatoma hyemalis* | DAhy | 0.58 | 0.018 |
|  | *Diatoma mesodon* | DIme | 0.71 | 0.005 |
|  | *Encyonema ventricosum* | ENve | 0.55 | 0.029 |
|  | *Eunotia catalana* | EUca | 0.79 | 0.009 |
|  | *Eunotia nymanniana* | EUny | 0.74 | 0.002 |
|  | *Fragilaria microvaucheriae* | FRmi | 0.52 | 0.040 |
|  | *Frustulia saxonica* | FRsa | 0.48 | 0.032 |
|  | *Gomphonema exilissimum* | GOex | 0.61 | 0.014 |
|  | *Hannaea arcus* | HNar | 0.67 | 0.006 |
|  | *Meridion circulare var. constrictum* | MDci | 0.66 | 0.008 |
|  | *Nitzschia alpina* | NIal | 0.73 | 0.002 |
|  | *Pinnularia pseudogibba* | PIps | 0.67 | 0.014 |
|  | *Planothidium lanceolatum* | PLla | 0.56 | 0.024 |
|  | *Psammothidium daonense* | PSda | 0.46 | 0.043 |
|  | *Ulnaria biceps* | ULbi | 0.79 | <0.001 |

**Supplementary figure 4** Co-occurrence networks of prokaryotic, eukaryotic and diatom communities from Núria and Chistau of different stream categories based on SparCC correlations > 0.6. Networks consist of nodes (dots, representing OTU’s) and edges (lines, representing interactions). Microbial network density (D) is indicated under each network. Diatom networks and density data for Chistau are not included due to insufficient data

**Supplementary Table 4** Diatom indicative species following the Indicator Value Method (p value <0.05, 9999 permutations) although not being significant

| ***Stream category*** | ***IndVal*** | ***p-value*** | ***Species*** |
| --- | --- | --- | --- |
| Acidic | 66.59 | 0.091 | *Fallacia vitrea* |
|  | 66.59 | 0.091 | *Fragilaria capucina* |
|  | 66.59 | 0.091 | *Gomphonema hebridense* |
|  | 66.59 | 0.093 | *Pinnularia pseudogibba* |
